# Electroantennogram reveals a strong correlation between the passion of honeybee and the properties of the volatile

**DOI:** 10.1002/brb3.1603

**Published:** 2020-04-09

**Authors:** Jieliang Zhao, Zhiqiang Li, Zhen Zhao, Yunqiang Yang, Shaoze Yan

**Affiliations:** ^1^ State Key Laboratory of Tribology Division of Intelligent and Biomechanical Systems Department of Mechanical Engineering Tsinghua University Beijing China; ^2^ School of Mechanical Engineering Beijing Institute of Technology Beijing China; ^3^ School of Engineering and Technology China University of Geosciences (Beijing) Beijing China

**Keywords:** electroantennogram, honeybee, olfactory recognition, passion

## Abstract

**Introduction:**

Insects use their antennae to detect food, mates, and predators, mainly via olfactory recognition of specific volatile compounds. Honeybees also communicate, learn complex tasks, and show adaptable behavior by recognizing and responding to specific odors. However, the relationship between the electroantennogram and the passion of honeybee has not been determined.

**Methods:**

We established a four‐channel maze system to detect the degree of sensitivity of the honeybee's antenna to different odors. In addition, electroantennography (EAG) signal was recorded from the right antennae of the honeybees in our experiments to explore electrophysiological responses to different volatiles.

**Results:**

The olfactory sensilla on the antennae of honeybees engender distinct electrophysiological responses to different volatiles. The bees were exposed to honey, 1‐hexanol and formic acid, and EAG parameters like depolarization time, falling slope, and amplitude were measured. The EAG indicators varied significantly between honey and formic acid, indicating either “happy” or “anxious” moods.

**Conclusions:**

Honeybee can express its passion by the characteristic changes of EAG parameters. We defined a preference factor (*F*) to quantify the preference of bees to varying concentrations of different compounds, where greater positive values indicate an increased passion. Our findings provide novel insights into the understanding of odor recognition in insects.

## Graphical abstract

Honeybee, as a eusocial flying insect, can also express its passion, even though there are no facial muscles on their face. We extract the characteristic physical quantities of EAG signals and analyze the response of bee antenna under different odors and different concentrations. The antenna potential of a honeybee, just like human facial expression, can reflect mood changes.

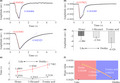

## 1 INTRODUCTION

Insects depend on the detection of volatile compounds through their specialized olfactory sensilla for basic functions like foraging, predation, and mate recognition (Haverkamp, Hansson, & Knaden, 2018; Pellegrino, Steinbach, Stensmyr, Hansson, & Vosshall, 2011; Pfeiffer, Ruther, Hofferberth, & Stökl, 2018; Ramdya et al., 2015). Highly eusocial insects like honeybees also communicate, learn complex tasks, and show adaptable behavior by recognizing and responding to specific odors (Leitch, Anderson, Paul Kirkbride, & Lennard, 2013; Missbach et al., 2014). Honeybee antennae are covered with small, tactile hairs that relay mechanosensory and gustatory information (Scheiner, Schnitt, & Erber, 2005) with more than 50% of these sensilla being for olfaction. Each sensillum placodeum is formed by an oval‐shaped, thin cuticular plate (9 μm × 6 μm) that has numerous minute pores and is innervated by 5–35 olfactory response neurons (ORNs).

Electroantennography (EAG) is a technique used to study the olfactory pathway in insects and was established by Schneider (1957). This technique measures the output of an insect antenna to its brain for a given odor. The response of an antenna to a volatile compound varies depending on the time of the day because the olfactory response and the pulse‐tracking ability of the antenna follows the circadian rhythm of the honeybee (Nagari, Szyszka, Galizia, & Bloch, 2017). There are also species‐specific variations in the responsiveness of antennae to the same odor; for example, *Apis cerana* consistently exhibits weaker antennal responses to the mandibular pheromone of the queen bee compared to the antennal responses of *Apis mellifera* (Dong et al., 2017). The EAG responses of honeybee antennae to varying concentrations of six common plant volatiles were previously evaluated, which showed that these responses can be utilized as odor probes (Bhowmik, Lakare, Sen, & Bhadra, 2016; Lambin, Déglise, & Gauthier, 2005). Honeybees, like vertebrates, respond to negative stimuli (Bateson, Desire, Gartside, & Wright, 2011) both physically and physiologically, which can be used as indicators of their emotions (Paul, Harding, & Mendl, 2005). Several physical behaviors, such as voluntary attention, memory, environmental perception, environmental judgment, anticipated effort, and responsibility (Lerner & Keltner, 2000), are recognized indices of emotion in a wide range of animal species. In addition, the intensity and duration of a tapered stimulus are the primary characteristics of sentiment‐driven behaviors (Anderson & Adolphs, 2014; Zhao, Yan, & Wu, 2016). Perry, Baciadonna, and Chittka (2016) found that bumblebees entered a positive, dopamine‐dependent emotional state upon receiving an unexpected reward of sucrose solution. Although honeybees have the ability to identify substances and express emotions, the relationship between the two remains unclear.

The aim of this study was to analyze the response of honeybees to various volatiles using EAG and a behavioral maze test in order to find a possible correlation between olfactory recognition and passion. In this study, the passion of honeybees in response to different volatiles was evaluated in terms of electroantennal physiology by behavioral and EAG experiments. While the former helped rank the smells in order of preference, the latter enabled the quantification of the characteristics of responses to the smells as measured by EAG, including amplitude, depolarization/repolarization time, and slope of rising/falling. In addition, a preference factor was calculated that can be used to objectively interpret the passion of honeybee in response to specific stimuli.

## 2 MATERIALS AND METHODS

### 2.1 Honeybee specimens

The honeybees (*Apis mellifera* L.) that were used in all of the experiments were obtained from the Tsinghua University of Beijing, China (40°N, 116°E). The temperature and humidity of the rearing room were maintained at 22–26°C and 40%–60%, respectively. The honeybees were settled down in a wooden hive and were free to enter a transparent glass box in the bottom of the beehive. The glass box was convenient for observing the behavior of foragers.

### 2.2 Chemicals

The test reagents used in the EAG experiments included plant volatile organic compounds and common chemicals. The purity, suppliers, and physical properties of the compounds are listed in Table 1.

**Table 1:** Basic information of four test reagents

Name	Purity	Suppliers	Properties/Significance
1‐Hexanol	98%	Macklin	It is regarded as the standard stimulus with special fragrance
Paraffin oil	AR	Sangon	It is colorless and odorless mineral oil, often used as organic solvent
Honey	≥99%	MH	It is a golden viscous liquid with sweet smell
Formic acid	≥88%	Rhawn	It's a colorless liquid with strong volatility and pungent odor

Abbreviation: MH, Manuka Health.

### 2.3 Electroantennography

The amplitude of the EAG signal was recorded from the right antennae of the honeybees in our experiments because the EAG responses of the right antennae triggered by the test reagents were palpably higher than those of the left antennae. The antennae of the honeybees were rapidly stabilized by incubating the honeybees at a low temperature (0°C) for approximately 3 min prior to starting the experiment. The stimulation airflow controller (Syntech, CS‐55) for the EAG experiment can convey two kinds of airflow, a steady airflow and a pulse airflow. Some of the test reagents were selected based on previous work (Bhowmik et al., 2016). In these experiments, the concentration of each reagent varied from 0.1 to 100 μl/ml. 1‐Hexanol was used as a reference standard and paraffin oil was used as a positive control. In each trial, we transferred 15 μl of the test compound to filter paper by using a Pasteur pipette. In addition, a sanitary napkin was placed under the filter paper to prevent the test solution from contaminating the antennae. The antenna was placed 5 mm away from the outlet of the plastic tube. The EAG signal measurement system was placed in a rectangular box (450 × 550 × 550 mm), which effectively reduced the interference caused by external vibration, air flow, and sound. The bee antennae were each fixed and connected to two electrodes. One electrode (Syntech, PRG‐3) was spliced with the upper end of the antenna by using conductive gel (Medtronic, Signa Gel) and the other electrode was connected to the lower end of the antenna, as shown in Extended Data Figure 1. An electron microscope was used to aid in the operation of joining the antenna to the electrodes.

**Figure 1:**
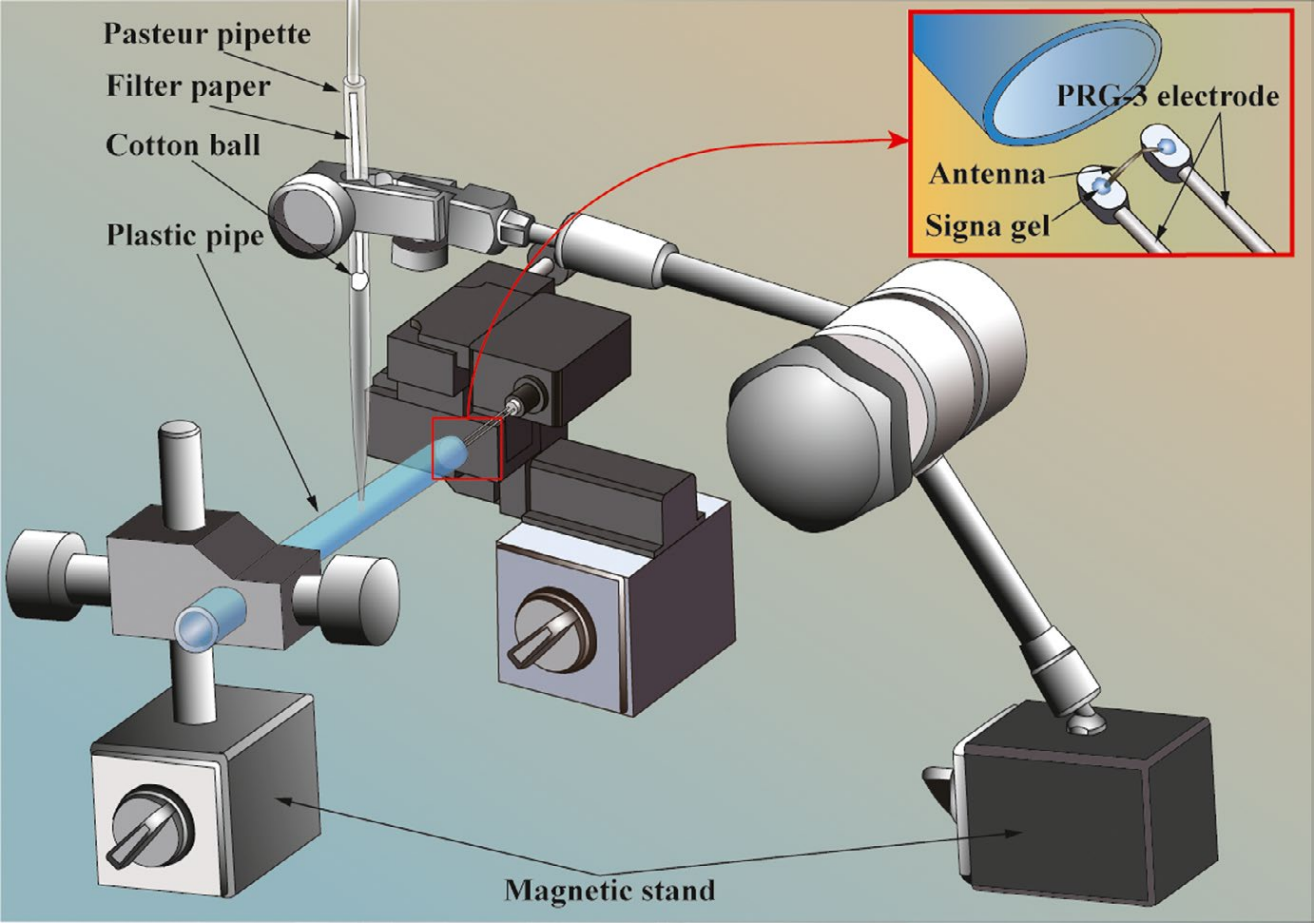
The components of the EAG system and the connection positions between the electrodes and the antennae

### 2.4 Behavioral experiments

A four‐channel maze system was designed to detect the degree of sensitivity of the honeybee's antenna to different odors. This system was composed of a four‐channel maze (with a radius of 10 cm, four channels with a width and height of 2 cm, and a central region that was a regular octagon with a side length of 2 cm), an odor source (containing volatile reagents), a two‐degree‐of‐freedom robotic arm, and a camera. When the bee's body reached the odor source, it was considered to be a valid time of access. Otherwise, it was considered to be an invalid access and was not counted in the cumulative number of times. Using this four‐channel maze system, four repetitive experiments were carried out and the bees from each trial were never reused. Initially, three different kinds of volatile reagents were injected into three difference odor sources, which had a layer of gauze on the top to prevent the bees from entering the solutions in the odor source. An odor source without a volatile reagent served as a control. Then, the four different odor sources were put into the four different channels and finally put back into the rectangular baffle. Subsequently, 10 bees entered from the circular hole at the center of the maze and then the round baffle was moved to cover the circular hole. The camera was used to record the cumulative times the bees accessed the different odor sources. The order of cumulative access times of the bees to the different odors is the preference order of the bees to the different odors. Supplementary experiments with only one odor source in the maze are necessary to determine the preference order if the bees have the same or very close cumulative access times to different kinds of odor sources and then the remaining steps are the same as above. The maze only has four channels because too many choices for the bees would result in the access times being very close for different odors, which would make the results of the experiment be of little significance.

## 3 RESULTS

**Figure 2:**
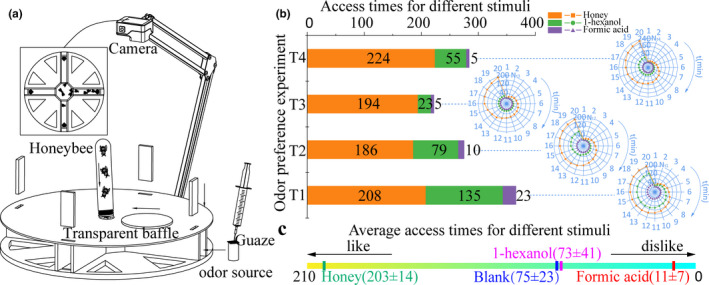
The preference of honeybees to different volatiles. (a) The schematics of the four‐channel maze system. (b) A stacked bar chart showing the number of visits toward the different compounds and a radar chart showing the time‐dependent variations in the number of visits. (c) The average number of visits for each odor from four tests

To prevent the aggregation of the honeybees, the maze pedestal was heated to 30 ± 1°C and three channels were filled with 0.4 ml of either honey, 40% of 1‐hexanol, or 40% of formic acid. The remaining channel was kept free of any odor. The four‐channel maze is shown in Figure 2a. For each test, the total number of visits to each odor source for 10 honeybees was recorded for 20 min. As shown in Figure 2b, the number of times the bees moved toward honey, 1‐hexanol, or formic acid was 208, 135, and 23 (N_t1_), respectively, in test one (T1). In T2, the number of times the bees moved toward honey, 1‐hexanol, or formic acid was 186, 79, and 10 (N_t2_), respectively. In T3, the number of times the bees moved toward honey, 1‐hexanol, or formic acid was 194, 23, and 5 (N_t3_), respectively. Finally, in T4, the number of times the bees moved toward honey, 1‐hexanol, or formic acid was 224, 55, and 5 (N_t4_), respectively. The average number of visits made in 20 min toward honey, the blank channel, 1‐hexanol, or formic acid were 203 ± 14, 75 ± 23, 73 ± 41, and 11 ± 7, respectively (Figure 2c), which clearly indicates that the honeybees preferred honey and were repulsed by formic acid.

## 4 DISCUSSION

**Figure 3:**
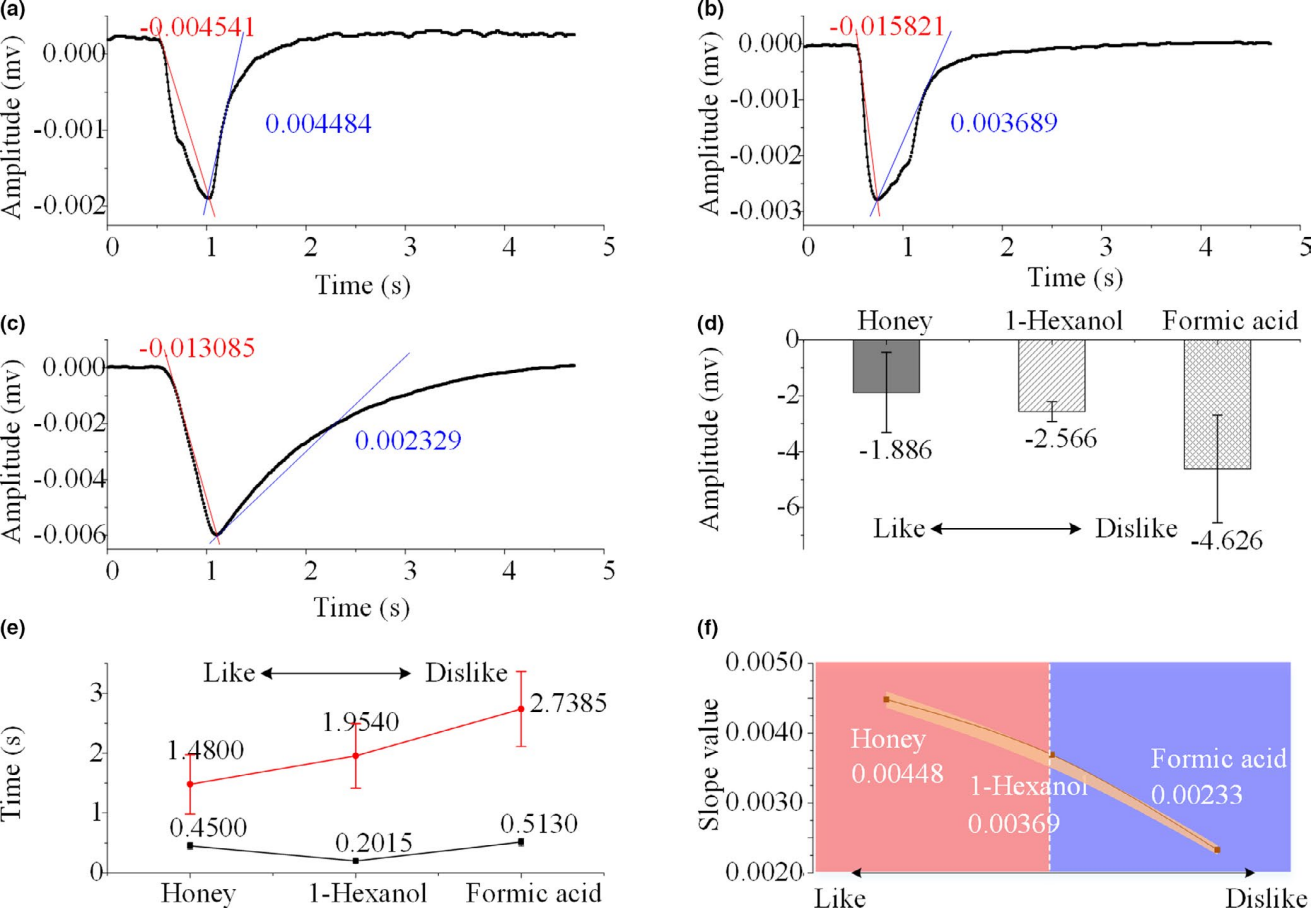
The EAG responses of *Apis mellifera* antennae to different compounds. (a–c) The average slope of the EAG responses to different compounds (honey/1hexanol/formic acid). The falling slopes (red line) and the rising slopes (blue line) are displayed. (d) The amplitudes of the EAG response elicited by varying concentrations of each compound. The sequence of compounds from left to right indicates the preference of the honeybees from “like” to “dislike”. (e) The depolarization and repolarization times for the different compounds. (f) The slope values of the EAG responses evoked by different compounds

Electroantennography (EAG) was then performed on 125 excised antennae that were exposed to the aforementioned volatiles and five parameters of the electrophysiological signals were evaluated. As shown in Figure 3(a–c), the absolute values of the amplitude, the falling slope and the depolarization time increased for all three volatiles at a concentration of 100 μl/ml. In addition, the rising slope was also elevated for honey and 1‐hexanol with increasing concentrations. However, the EAG rising slope increased steadily for formic acid at concentrations from 0.1 to 10 μl/ml, but plateaued at concentrations between 10 and 100 μl/ml. The repolarization time did not change significantly as a function of concentration for any of the volatiles (as shown in Appendix S1), but there might be individual differences across the antennae.

To further clarify the relationship between honeybee passions and the EAG responses, the relative amplitude, time, and slope of the EAG signals were compared and analyzed (Yang, Zhang, Gurr, Vasseur, & You, 2016). As shown in Figure 3(d–f), the relative amplitudes of the EAG responses increased from honey to formic acid, as did the relative depolarization times from honey to formic acid. The relative falling slopes also increased across the gradient from honey to formic acid. When the honeybees were in a “happy” mood, the relative amplitudes varied from 0.33 to 0.75, the relative depolarization times varied from 1.65 to 2.23, and the relative falling slopes varied from 0.25 to 0.38. When the honeybees were in an “anxious” mood, the range of the relative amplitudes was 0.43–1.80, the range of the relative depolarization times was 1.80–2.55, and the range of the relative falling slopes was 0.30–0.83. In contrast, the repolarization times and the rising slopes showed considerable individual variations making them not indicators of honeybee passion. Based on our results, we defined a preference factor (*F*) to determine the preference of honeybees to different volatiles as follows:

(1) $$F\left(x\right)=\sum_{c\;=\;0.1}^{100}\left(\frac{A_{c}\cdot e^{\frac{c}{k}}}{\left(T_{c}\cdot S_{c}\right)}\right)$$

where *x* refers to a compound, *c* is the concentration of the compound that varies from 0.1 to 100 μl/ml, *A_c_* is the amplitude of the EAG response elicited by the compound at the specific concentration, *k* is the modified constant 10, *T_c_* is the depolarization time and *S_c_* is the falling slope. Accordingly, the *F* values for honey, 1‐hexanol, and formic acid were 2.04 × 10^7^, 1.79 × 10^7^, and 1.53 × 10^7^, respectively, indicating a similar order of preference as in the behavioral experiments.

The olfactory sensilla of honeybee antennae function as organs of passion by responding to the surrounding odors. The behavioral test showed that the honeybees responded most favorably to honey and were repulsed by formic acid. The “happy” or “anxious” moods in response to the different odors were measured in terms of the EAG amplitudes, depolarization, and repolarization times, and the rising and falling slopes. From these data, the preference factor (*F*) was calculated as a measure of odor preference. Our findings provide new insights into the odor recognition of insects.

## CONFLICT OF INTEREST

The authors declare no conflict of interests.

## AUTHOR CONTRIBUTIONS

J. Z., Z. L., and Z. Z. performed the experiments. J. Z. and Z. L. analyzed the data and wrote the manuscript. S. Y. and Y. Y. conceptualized the work and critically revised the manuscript. All authors read and approved the final version of the manuscript.

## ETHICAL APPROVAL

Not required.

## Supporting information

Supplementary Material

## Data Availability

The data that support the findings of this study are available from the corresponding author upon reasonable request.
