# Supplementary material for: Electroantennogram reveals a strong correlation between the passion of honeybee and the properties of the volatile
Source: Brain Behav. 2020 Apr 9;10(6):e01603. doi: 10.1002/brb3.1603 (PMC7303395; doi:10.1002/brb3.1603)
Supplement: Supplementary file 1 — Supplementary Material [file BRB3-10-e01603-s001.doc]

**Electroantennogram reveals a strong correlation between the passion of honeybee and the properties of the volatile**

**Running Title:** Electroantennogram is the mirror of honeybee passion

**Jieliang Zhao^1,2+^*, Zhiqiang Li^3+^, Zhen Zhao^3^, Yunqiang Yang^3^ and Shaoze Yan^1^***

^1^Division of Intelligent and Biomechanical Systems, State Key Laboratory of Tribology, Department of Mechanical Engineering, Tsinghua University, Beijing, 100084, P. R. China

^2^School of Mechanical Engineering, Beijing Institute of Technology, Beijing, 100081, P. R. China

^3^School of Engineering and Technology, China University of Geosciences (Beijing), Beijing, 100083, P. R. China

+These authors contributed equally to this study.

*Correspondence:

1. Shaoze Yan, Room A1032, Lizhaoji Building, Tsinghua University, Beijing 100084, P. R. China

Email: [yansz@mail.tsinghua.edu.cn](mailto:yansz@mail.tsinghua.edu.cn) Ph: +86-10-6279-6046 Fax: +86-10-6279-6046

2. Jieliang Zhao, Room 303, No.1 Building, Beijing Institute of Technology, 5 South Zhongguancun Street, Haidian District, Beijing 100081, P. R. China

Email: [jielzhao@bit.edu.cn](mailto:jielzhao@bit.edu.cn) Ph: +86-13522365096

**Supplementary Information**

## Behavior experiment

To determine the susceptibility of honeybee's antenna to different odors, a four-channel maze was designed. The system mainly includes a four-channel maze (with a radius of 10 cm, four corridors with a width and height of 2 cm, and a central region of regular octagon with a side length of 2 cm), an odor source (containing volatile reagents) in the end of each maze corridor, a two-degree-of-freedom robotic arm, and a camera (GoPro Hero6 Black, GoPro, USA). When a honeybee reaches the destination with odor source, it can be viewed as an efficient count for direct access to his favorite substance. Otherwise, it is an invalid access and was not counted in the visit frequency. In our research, four repetitive experiments were carried out. The honeybees for each trial were never reused. At first, three different kinds of volatile reagents were injected into the odor source of three maze corridors, all of which had a layer of gauze on the top to prevent the honeybees from taking in too much solution. The rest odor source without the volatile reagent was used as the reference category. So that, four different odor sources were put into four corridors of the maze respectively. Furthermore, the rectangular baffles were put back in the maze. Afterwards, 10 honeybees entered from the circular hole of the maze center. And then, the round baffle was moved to cover the circular hole. Finally, the GroPro camera was used to record the visit frequency of different odor sources accessed by the honeybees. The likability rating of different odors for honeybee can be ordered by priority in the result of visit frequencies. Supplementary experiments with only one odor source in the maze are necessary if honeybees have the same or very close visit frequency on two of these kinds of odor sources. The remaining steps are the same as above. If there are too many choices for the honeybees, causing their visit frequencies very close to each other, the results of the experiments should be of little significance.

## Statistical analysis

All statistical analyses were calculated by using the statistical software SPSS 24.0 (SPSS Inc., Chicago, IL, USA). Furthermore, the Student’s *t*-test was employed to examine significant differences in the potential characteristics of antenna between live and excised groups. Multiple comparisons were made and calculated as least square differences. Two-way analysis of variance was used to test the difference in excised antenna between the three volatiles with different concentrations. All values are shown as means ± s.e.m. unless noted otherwise.

As shown in Fig. S1, the absolute value of amplitude, the falling slope and the depolarization time increased for all three volatiles in a concentration-dependent manner. In addition, the rising slope was also elevated for honey and 1-hexanol with increasing concentration. In case of formic acid however, the EAG rising slope increased steadily at concentrations from 0.1 to 10μl/ml, but plateaued between 10-100μl/ml. The repolarization time did not change significantly as a function of concentration for any of the volatiles. However, there might be individual differences across the antennae.

| 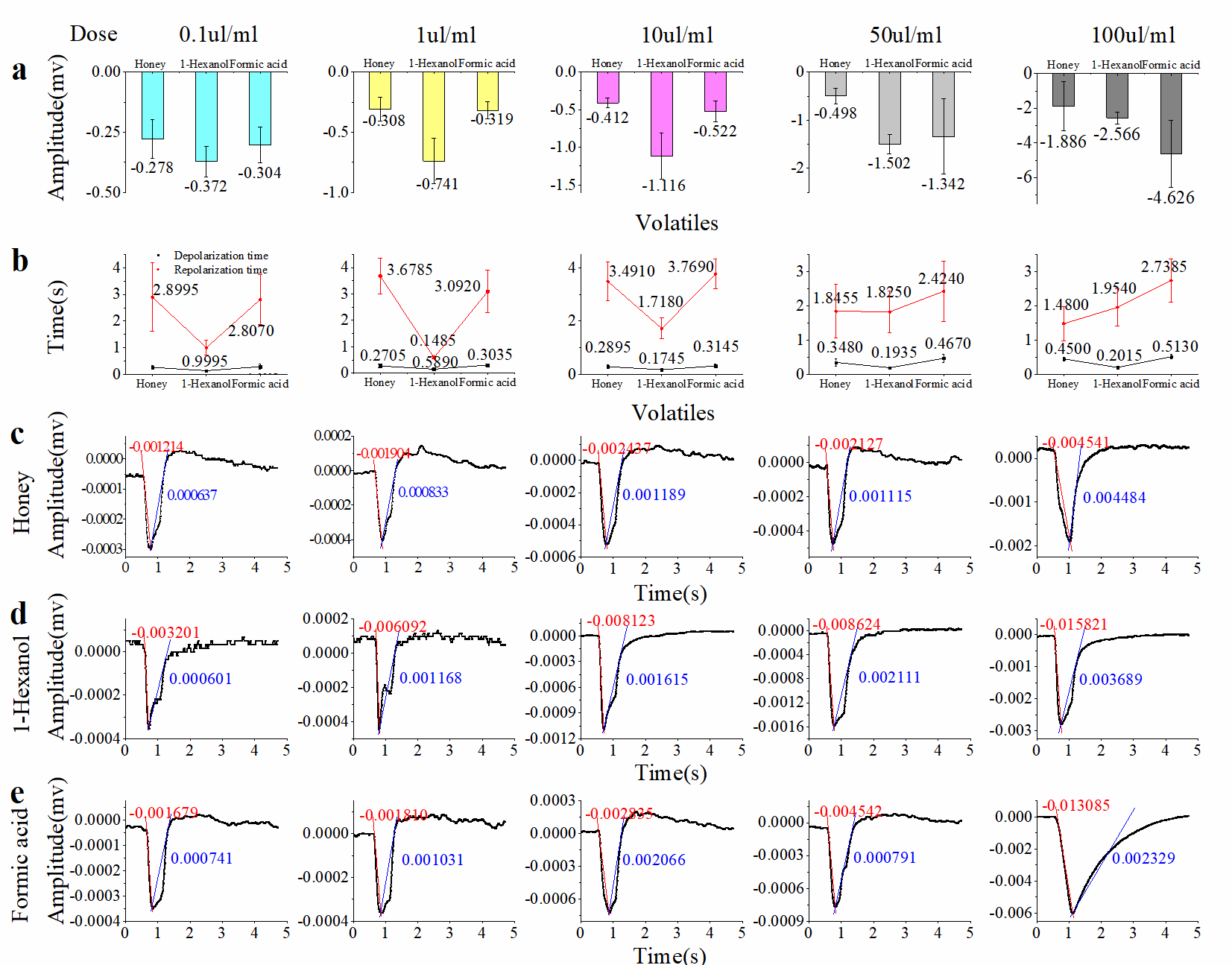 |
| --- |
| **Extended Data Figure S1.** **The EAG responses of honeybee antennae for different compounds in a concentration-dependent manner. (a) The amplitude of EAG in response to varying concentrations of each compound; the sequence of compounds from left to right indicates the preference of the honeybees from “like” to “dislike”. (b) The depolarization and repolarization times for the different compounds. (c-e) Average slope of EAG response to varying concentrations of honey/1hexanol/formic acid. Falling slope (red line) and rising slope (blue line) are displayed.** |

As shown in Fig. S2, the heatmap indicates the EAG responses of honeybees changing along a concentration gradient of different compounds. With the concentration of 0.1μL/mL, the amplitude of the EAG to 1-hexanol was significantly lower than that of honey(p<0.001) and formic acid(p=0.004), but there was no significant difference between honey and formic acid(p=0.272). The depolarization and repolarization time of the EAG to 1-hexanol was significantly lower than that of honey(p<0.001) and formic acid(p<0.001), but there was no significant difference between honey and formic acid(p>0.05). Similarly, the falling slope of the EAG to 1-hexanol was significantly lower than that of honey(p<0.001) and formic acid(p<0.001). And the falling slope of the EAG to formic acid was significantly lower than that of honey (p=0.032). The rising slope of the EAG to 1-hexanol was significantly lower than that of formic acid (p=0.014), but there was no significantly difference between honey and others (p>0.05). With the concentration of 1μL/mL, the amplitude of the EAG to 1-hexanol was significantly lower than that of honey(p<0.001) and formic acid(p<0.001), but there was no significant difference between honey and formic acid(p=0.789). The depolarization time of the EAG to 1-hexanol was significantly lower than that of honey(p<0.001) and formic acid(p<0.001), and the depolarization time of the EAG to honey was significantly lower than that of formic acid(p=0.043). The repolarization time of the EAG to 1-hexanol was significantly lower than that of honey(p<0.001) and formic acid(p<0.001), and the repolarization time of the EAG to formic acid was significantly lower than that of honey(p=0.003). The falling slope of the EAG to 1-hexanol was significantly lower than that of honey(p<0.001) and formic acid(p<0.001), but there was no significant difference between honey and formic acid(p=0.760). The rising slope of the EAG to honey was significantly lower than that of formic acid(p=0.005) and 1-hexanol(p<0.001), and the rising slope of the EAG to formic acid was significantly lower than that of 1-hexanol(p=0.047). With the concentration of 10μL/mL, the amplitude of the EAG to 1-hexanol was significantly lower than that of honey(p<0.001) and formic acid(p<0.001), but there was no significant difference between honey and formic acid(p=0.084). The depolarization time of the EAG to 1-hexanol was significantly lower than honey(p<0.001) and formic acid(p<0.001), but there was no significant difference between honey and formic acid(p=0.177). The repolarization time of the EAG to 1-hexanol was significantly lower than that of honey(p<0.001) and formic acid(p<0.001), but there was no significant difference between honey and formic acid(p=0.130). The falling slope of the EAG to 1-hexanol was significantly lower than that of honey(p<0.001) and formic acid(p<0.001), but there was no significant difference between honey and formic acid(p=0.500). The rising slope of the EAG to honey was significantly lower than that of formic acid(p<0.001) and 1-hexanol(p=0.003), and the rising slope of EAG to 1-hexanol was significantly lower than that of formic acid (p=0.002). With the concentration of 50μL/mL, the amplitude of the EAG to honey was significantly higher than that of 1-hexanol(p<0.001) and formic acid(p<0.001), but there was no significant difference between 1-hexanol and formic acid(p=0.290). The depolarization time of the EAG to 1-hexanol was significantly lower than that of honey(p<0.001) and formic acid(p<0.001), and the depolarization time of the EAG to honey was significantly lower than that of formic acid(p<0.001). The repolarization time of the EAG to formic acid was significantly higher than that of honey(p=0.020) and 1-hexanol(p=0.016), but there was no significant difference between honey and 1-hexanol(p>0.05). The falling slope of the EAG to 1-hexanol was significantly lower than that of honey(p<0.001) and formic acid(p<0.001), and the falling slope of the EAG to formic acid was significantly lower than that of honey(p=0.001). The rising slope of the EAG to formic acid was significantly lower than that of honey (p=0.002) and 1-hexanol(p<0.001), and the rising slope of the EAG to honey was significantly lower than that of 1-hexanol (p<0.001). With the concentration of 100μL/mL, the amplitude of the EAG to formic acid was significantly lower than that of honey(p<0.001) and 1-hexanol(p<0.001), but there was no significant difference between honey and 1-hexanol(p=0.131). The depolarization time of the EAG to 1-hexanol was significantly lower than that of honey(p<0.001) and formic acid(p<0.001), and the depolarization time of the EAG to honey was significantly lower than that of formic acid(p<0.001). The repolarization time of the EAG to honey was significantly lower than that of 1-hexanol(p=0.009) and formic acid(p<0.001), and the repolarization time of the EAG to 1-hexanol was significantly lower than that of formic acid(p<0.001). The falling slope of the EAG to honey was significantly higher than that of 1-hexanol (p<0.001) and formic acid(p<0.001), but there was no significant difference between 1-hexanol and formic acid(p=0.132). The rising slope of the EAG to formic acid was significantly lower than that of honey (p=0.010), but there was no significantly difference between 1-hexanol and others(p>0.05).

| 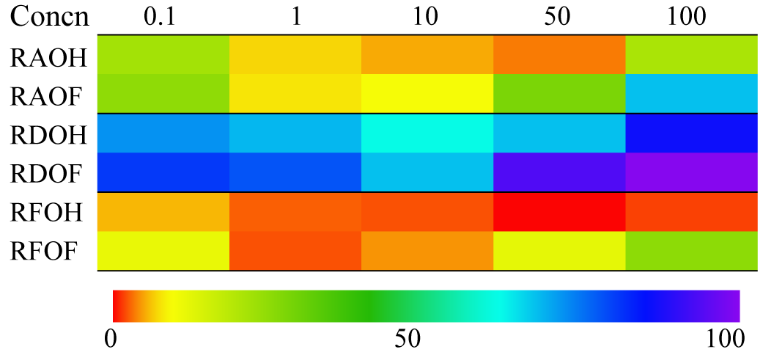 |
| --- |
| **Extended Data Figure S2. The heatmap of** **the EAG responses of honeybees to different compounds along a concentration gradient. RAOH,**  **the relative amplitudes of**  **honey; RAOF,**  **the relative amplitudes of**  **formic acid;**   **RDOH, the relative depolarization times of honey; RDOF, the relative depolarization times of formic acid; RFOH, the**  **relative falling slopes of honey; RFOF, the**  **relative falling slopes of formic acid.** |
